# Harnessing potassium peroxymonosulfate activation of WO_3_/diatomite composites for efficient photocatalytic degradation of tetracycline

**DOI:** 10.1039/d4ra04447a

**Published:** 2024-08-09

**Authors:** The Luan Nguyen, Hong Huy Tran, Tu Cam Huynh, Khoa Tien Le, Thi Minh Cao, Viet Van Pham

**Affiliations:** a University of Science, VNU-HCM 227 Nguyen Van Cu Street, District 5 Ho Chi Minh City 700000 Vietnam; b Vietnam National University Ho Chi Minh City Ho Chi Minh City 700000 Vietnam; c Advanced Materials and Applications Research Group (AMA), HUTECH University 475A Dien Bien Phu Street, Binh Thanh District Ho Chi Minh City 700000 Vietnam pv.viet@hutech.edu.vn

## Abstract

The increasing prevalence of pharmaceutical contaminants in aquatic ecosystems poses profound challenges for both environmental sustainability and public health. Addressing this pressing issue requires the development of innovative, cost-effective, and efficient remediation approaches. Here we report the synthesis of WO_3_/diatomite composites and their photocatalytic degradation in conjunction with potassium peroxymonosulfate (PMS) activation. By leveraging the synergistic effects, we observe a remarkable degradation of tetracycline, a significant pharmaceutical contaminant, under visible light. Analytically, we have elucidated the driving factors for the enhanced performance, emphasizing the optimal amount of WO_3_ (10%) in the composite and PMS concentration (3 mM). Specifically, the WO_3_/diatomite catalyst presents a degradation rate of 80.75% tetracycline (40 mg L^−1^) after 180 min of visible light irradiation. Also, we elucidate the primary roles of ˙SO_4_^−^ radicals in driving the photocatalytic reaction using free radical trapping studies. Our approach not only offers a direct solution to controlling pharmaceutical contamination but also opens new possibilities for advancing the design of composite-based photocatalysts by taking advantage of nature-derived materials.

## Abstract 2

The increasing prevalence of pharmaceutical contaminants in aquatic ecosystems poses profound challenges for both environmental sustainability and public health.

## Graphical abstract

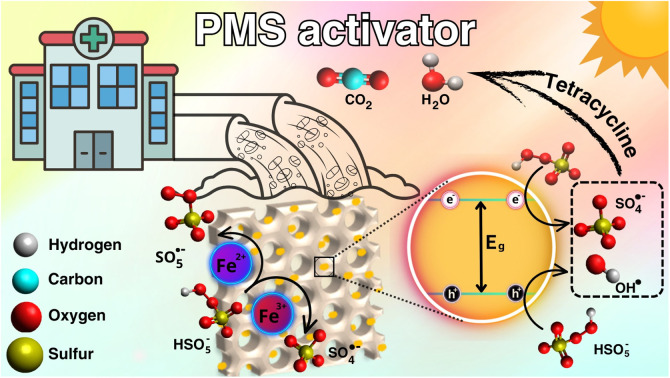

## 1. Introduction

While industrial progression has improved human living standards, it has concurrently posed environmental challenges.^1,2^ Notably, the emerging healthcare sector discharges a considerable volume of pharmaceutical pollutants. Specifically, as an effective antibiotic in treating various bacterial infections, for instance, tetracycline is one of the most widely used antibiotics globally and is a predominant aquatic pharmaceutical pollutant.^3–5^ Tetracycline possesses a highly stable chemical structure. When it is administered into the body through injection or oral delivery, only 20% is absorbed and metabolized, which in turn suggests that the remaining 80% is discharged. Human health may be at risk from antibiotic residues found at values ranging from 0.4 ng L^−1^ to 35.5 μg L^−1^.^6^ Undoubtedly, addressing pharmaceutical pollution is imperative in maintaining our well-being.

The conventional approaches such as filtration, adsorption, and biodegradation fail to effectively degrade the antibiotics given their high stability and low biodegradability.^7–10^ Extensive research underscores the notable effectiveness of advanced oxidation processes (AOPs), particularly photocatalysis, as a prime solution for tackling antibiotic pollution. The AOPs are not only an environmentally friendly process for effective treatment of antibiotics but also curtails the emergence of antibiotic-resistant bacteria in water systems, thereby addressing a major global health challenge.^11^

Standing out from various photocatalytic materials, tungsten oxide (WO_3_) materials are particularly well-suited as a photocatalyst for tackling antibiotic pollution because of their exceptional stability and strong absorption of visible light (*E*_g_ ∼2.7–3.3 eV), which allows for efficient use of solar energy.^12–15^ The synthesis of WO_3_ materials is well documented that offers a distinct advantage in terms of potential scalability and cost-effectiveness for future manufacturing.^16,17^ Additionally, their non-toxic characteristics position them a potentially safe and sustainable option for water treatment applications, ensuring minimal environmental footprint while combating this urgent concern.^18,19^ Nonetheless, it is important to note that WO_3_ photocatalysts also present unmet limitations. WO_3_ has a relatively narrow band gap (*E*_g_), which means it is prone to rapid charge recombination. The photocatalytic degradation of WO_3_ may require extended reaction times compared to other photocatalysts, compromising the time efficiency of the photocatalytic degradation. Also, WO_3_ materials can be susceptible to photocorrosion, especially under prolonged exposure to light, potentially diminishing their photocatalytic performance and overall stability.^20,21^ These fundamental limitations underscore the imperative for continued research aimed at optimizing WO_3_-based photocatalysis for efficient antibiotic removal from water sources.

To overcome these challenges and maximize its efficiency, the creation of appropriate composites is emerging as an innovative solution. Notably, diatomite, a naturally occurring known as diatomaceous earth or bio-silica, has recently emerged as a compelling contender for augmenting the photocatalytic activity.^22–25^ Comprised of the fossilized remains of diatoms, tiny aquatic plants, its unique physicochemical properties provide a solid foundation for various environmental applications, including water purification.^22^ Specifically, diatomite, a remarkable sedimentary rock primarily composed of silica microfossils derived from aquatic unicellular algae, boasts a suite of distinctive attributes, including its high porosity (ranging from 25% to 65%), small particle dimensions, expansive surface area, robust adsorption capacities, and natural abundance and cost-effectiveness.^26–28^ Furthermore, diatomite demonstrates versatility as a catalyst support and a reservoir for essential diatomic species, such as molecular oxygen (O_2_) or hydrogen peroxide (H_2_O_2_), pivotal co-reactants in photocatalytic reactions.^22^

Concurrently, the use of potassium peroxymonosulfate (2KHSO_5_·KHSO_4_·K_2_SO_4_, PMS) activation emerges as a strategic approach to enhance the reaction kinetics of photocatalysts.^29^ Notably, PMS introduces highly reactive sulfate radicals (˙SO_4_^−^), precipitating rapid antibiotic oxidation and thus expediting pollutant removal.^30–32^ This process additionally offers the potential to ameliorate the issue of photocorrosion, with PMS actively promoting sulfate radical formation, thereby reducing catalyst degradation risks and enhancing stability.^20,33^ The rational combination of diatomite and PMS activation leverages these collective advantages, fostering a synergistic platform poised to amplify the catalytic activity of WO_3_, thus offering a promising pathway for more efficient antibiotic degradation within water treatment processes. Despite a remarkable advancement in developing WO_3_-based photocatalytic materials for degrading tetracycline, the complicated and high-cost synthesis of these materials remains an unmet hurdle. Also, these catalysts often present a broad band gap value, hindering their activity exclusively under UV radiation, which consequently limits the applicability of in living conditions.

Here we present the fabrication of a composite photocatalyst by anchoring WO_3_ onto diatomite using a straightforward mechanical mixing method. The resultant WO_3_-loaded diatomite materials demonstrate remarkable efficiency in removing tetracycline under visible light exposure in assistance of PMS. Our findings emphasize that the exceptional performance of WO_3_/diatomite composites, when activated with PMS, can be attributed to the synergistic interactions between these components. This innovative integration effectively addresses the inherent limitations of WO_3_, ultimately advancing its potential as a robust solution for combating antibiotic pollution, with promising implications for further scientific investigation and application. This study also lays the foundation for advancing the design of innovative photocatalysts with heightened performance and multifaceted capabilities.

## 2. Experimental

### 2.1. Fabrication of materials

WO_3_ nanoplates were synthesized through a hydrothermal approach. An initial solution was prepared by dissolving 4.125 g of Na_2_WO_4_·2H_2_O (Sigma-Aldrich, ≥99%) and 2.125 g of NaNO_3_ (Sigma-Aldrich, ≥99%) in 100.0 mL of distilled water. Subsequently, 60.0 mL of aqueous HNO_3_ (Xilong, China, 68% wt) was slowly added to the above solution, magnetically stirred for one day at room temperature. The resulting solution was then transferred to a 200 mL Teflon autoclave and subjected to a hydrothermal condition at 150 °C for one day, after which it was cooled naturally to room temperature. The resulting yellow precipitate was collected, thoroughly washed with distilled water, and subsequently dried at 80 °C for 120 minutes. Finally, the obtained powder was annealed in an air atmosphere at 400 °C for 120 minutes to produce WO_3_ nanoplates. The diatomite material was sourced from Phu Yen province, Vietnam, and underwent milling to produce a powder characterized by small and relatively uniform particle sizes.

**Scheme 1:**
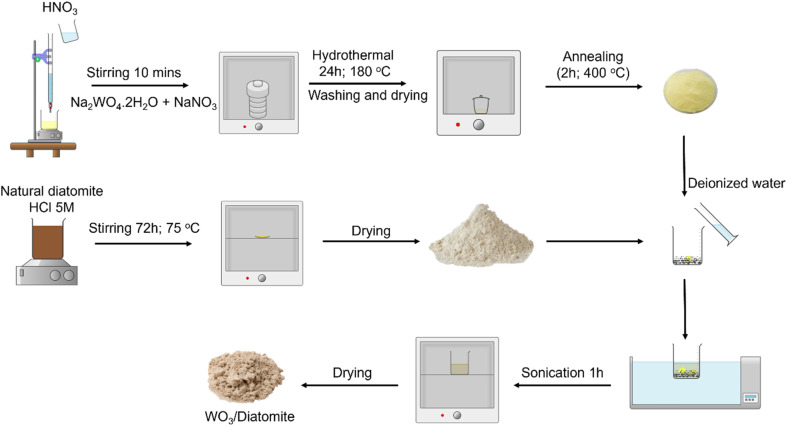
The overall fabrication process of WO_3_/diatomite composites.

The preparation of WO_3_/diatomite composites involved an ultrasonic process, varying the WO_3_ mass percentage at 3%, 5%, 10%, 30%, and 50% by weight. Initially, WO_3_ powder was introduced into a beaker containing a diatomite suspension in 20.0 mL of deionized (DI) water. The amalgamation was subsequently subjected to one hour of sonication followed by one hour of stirring. The resulting mixture was then dried for 240 minutes at 80 °C. Finally, the obtained powder was further processed by grinding using mortars and pestles. The overall process of the synthesis is schematically illustrated in Scheme 1.

### 2.2. Characterization techniques

Various analytical techniques were applied for a comprehensive characterization. To investigate the crystal structure, X-ray diffraction (XRD) patterns were analyzed using a Siemens/Bruker D5000 X-ray powder diffractometer system, employing Cu–Kα radiation (*λ* = 1.54064 Å). Chemical bonds in the materials were examined by capturing their characteristic molecular vibration using an FT-IR spectrophotometer (Jasco FT-IR–4700). The morphology of the samples was visualized through scanning electron microscopy (SEM) images (JMS-IT500). The elemental composition of the materials was assessed using energy-dispersive X-ray spectroscopy in conjunction with scanning electron microscopy (SEM-EDX, JEM 2100, JEOL). Optical properties were studied using UV-Vis diffuse reflection spectroscopy (DRS) on a Jasco V–770 UV/Vis spectrophotometer. Details about the material's conduction band were studied through an electrochemical approach using the Mott–Schottky (MS) plot on three-electrodes Biologic SP200. Solution concentrations were analyzed using a UV-Vis spectrophotometer (Hitachi U-2910).

### 2.3. Experimental details of the photocatalytic degradation

This investigation aimed to evaluate the potential of WO_3_/diatomite composites to activate PMS (Acros Organics, 4.5% active oxygen) for the degradation of tetracycline under visible light. To determine the optimal material composition for achieving the highest photocatalytic efficiency, a systematic assessment of the photocatalytic performance was conducted, varying the WO_3_ concentrations in the WO_3_/diatomite composite. The reactions were carried out in a 100 mL glass beaker (7.2 cm in height and 5 cm in diameter, the distance from the lamp height to the solution is 12 cm) containing 60 mL tetracycline solution, which was dissolved from tetracycline hydrochloride 500 mg, with continuous magnetic stirring. Initially, to establish adsorption/desorption equilibrium, 12 mg of photocatalyst was introduced into the target tetracycline solution and stirred for 150 minutes in the absence of light. Subsequently, the suspension was illuminated for 180 minutes using a sunshine simulator lamp (Osram Ultra-Vitalux 300 W, 230 V) equipped with a UV cut-off filter (*λ* > 420 nm) and a cooling fan. To analyze the absorption profile, samples were centrifuged, placed in cuvettes, and subjected to UV-Vis monitoring at 30 minutes intervals for a total of 180 minutes. The effectiveness of the photocatalytic tetracycline degradation of the materials is quantified through eqn (1).^34^1
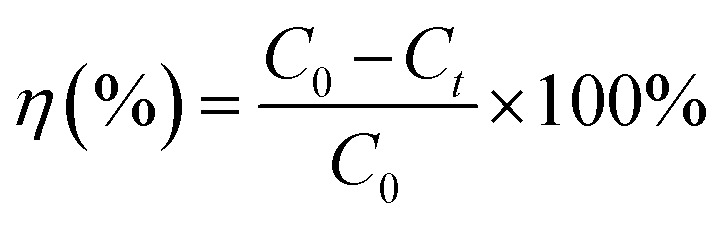
where *η* is photocatalytic degradation efficiency, *C*_0_ is the absorption intensity of the tetracycline solution after the adsorption/desorption reaches equilibrium, *C*_*t*_ is the absorption intensity of the solution at time *t*. The first order kinetic equation (eqn (2)) is used to get the reaction rate constant of the tetracycline decomposition.2
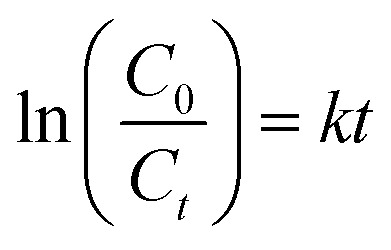
where *k* is the reaction rate constant of the ideal first-order equation (min^−1^). The plot of 
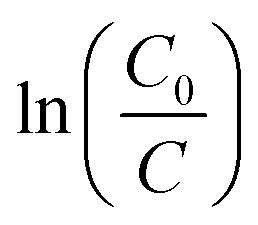
*versus t* gives a linear behavior, and *k* is calculated from its slope.

To empirically identify the dominant factor driving the photocatalytic activity, we conducted reactive species trapping tests.^35^ In these experiments, we introduced 1 wt% of specific scavengers – IPA, TBA, K_2_Cr_2_O_7_, KI, and BQ-to the photocatalyst, targeting ˙OH and ˙SO_4_^−^, ˙OH, e^−^, h^+^, and ˙O_2_^−^, respectively. The presence of scavengers was aimed at reducing the efficiency of photocatalytic performance, thereby elucidating the roles played by each type of free radicals during the photocatalytic activity.

## 3. Results and discussion

### 3.1. Structural characterizations

**Fig. 1:**
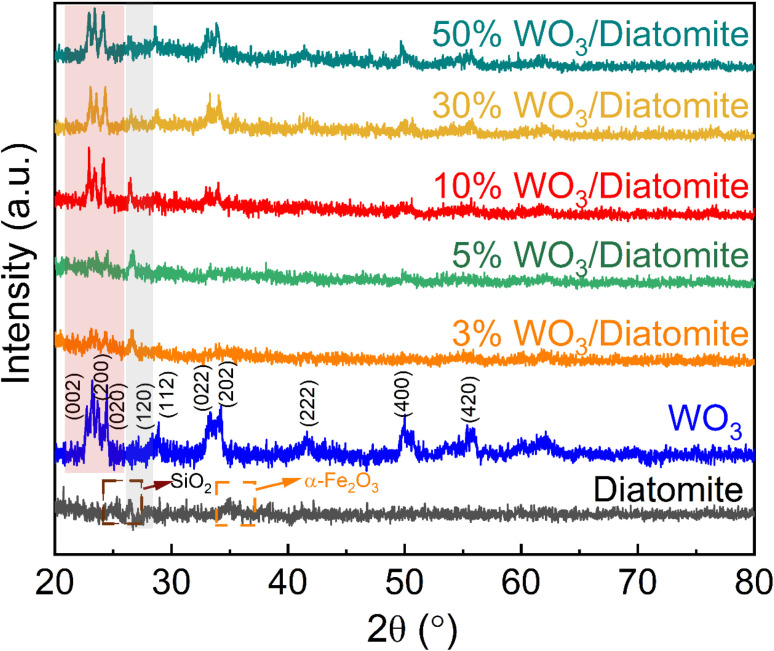
XRD patterns of diatomite, WO_3_, and WO_3_/diatomite composites.

The crystal structures of the diatomite, WO_3_, and WO_3_/diatomite composites with varying WO_3_ concentrations can be informed by their distinct X-ray diffraction peaks (Fig. 1). The characteristic diffraction peaks of WO_3_ materials are clear at 2*θ* diffraction angles of 23.15°, 23.50°, 24.53°, 27.16°, 28.91°, 33.33°, 34.46°, 41.62°, 50.01°, and 55.37°, associating with the lattice faces (002), (020), (200), (120), (112), (022), (202), (222), (400), and (420), planes of monoclinic, respectively [ICDD 83-0950]. These well-defined peaks underscore the crystalline nature of WO_3_ within the samples. The distinct peaks corresponding to diatomite are observed at 26.60° and 33.10°, featuring the coexistence of SiO_2_ and α-Fe_2_O_3_, respectively [JCPDS Card no. 13-0026]. These findings align with the elemental composition of naturally occurring diatomite as SiO_2_ and Fe_2_O_3_ constitute the majority of its composition.^28^ The 3%, 5%, and 10% WO_3_/diatomite samples exhibit peaks indicative of diatomite, confirming the dominant presence of diatomite within these composite materials. Interestingly, in the 30% and 50% WO_3_/diatomite composites, we also observe characteristic peaks of WO_3_, specifically corresponding to the lattice faces (002), (020), (200), (022), and (202). These XRD results provide critical insights into the structural composition of the composites, highlighting the coexistence of WO_3_ and diatomite.

**Fig. 2:**
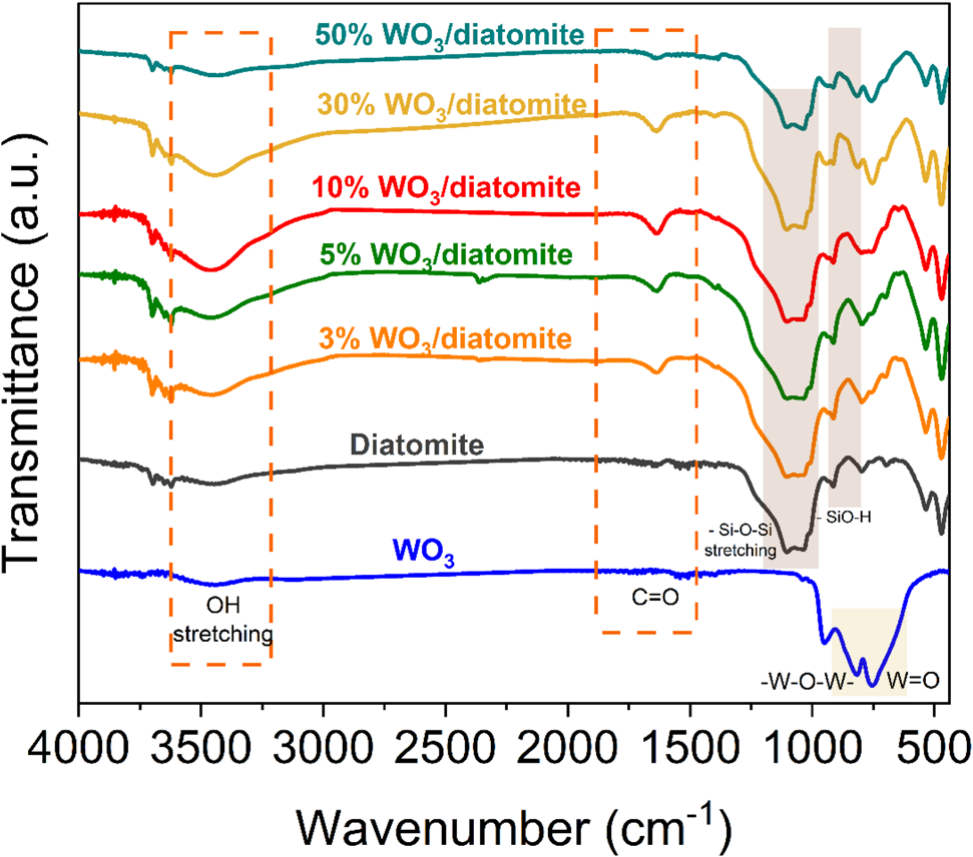
FTIR profiles of diatomite, WO_3_, and WO_3_/diatomite composites.

Furthermore, the specific chemical bonding vibrations of WO_3_, and WO_3_/diatomite composites were analyzed through FT-IR spectra (Fig. 2). In the FT-IR spectra of WO_3_, the discernible peaks spanning the range of 700 cm^−1^ to 900 cm^−1^ are attributed to the stretching vibrations of W=O and W–O–W bonds.^36^ Notably, these characteristic peaks of WO_3_ become more pronounced as the WO_3_ content in the composites increases, consistent with the trends observed in the XRD patterns. The pronounced peak at 1098 cm^−1^ is attributed to the stretching mode of siloxane (Si–O–Si) bonds, underlining the presence of these bonds in the materials.^37^ Additionally, the peak at 471 cm^−1^ corresponds to the asymmetric stretching mode of siloxane bonds.^38^ Furthermore, the vibration of O–H bonds contributes to the peak observed at 796 cm^−1^. It is worth noting that the broad absorption peak at 3442 cm^−1^ is associated with the stretching mode of the –OH group, stemming from water molecules absorbed on the material's surface. This adsorption can be attributed to the porous structure inherent in diatomite, which facilitates water adsorption. This comprehensive FT-IR analysis not only validates the materials' elemental composition in supporting the XRD results about the successful combination of WO_3_ and diatomite but also sheds light on their chemical characteristics and surface properties, further enriching our understanding of their potential applications in photocatalysis.

### 3.2. Morphological

**Fig. 3:**
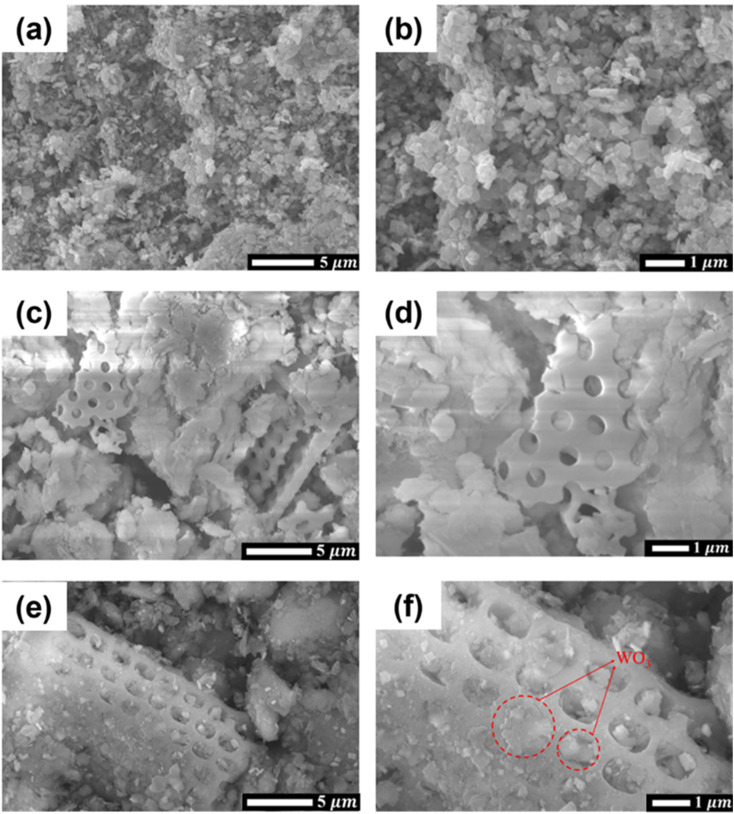
SEM images of (a and b) WO_3_ (c and d) diatomite, and (e and f) 10% WO_3_/diatomite composites.

Scanning electron microscopy (SEM) images are captured to visualize the materials' morphology. Fig. 3a and b vividly depicts the irregular arrangement of structural elements comprising WO_3_ nanoplates. The acid treatment's effect on diatomite's morphology is evident in Fig. 3c and d, revealing a noticeable enhancement in pore visibility and a substantial reduction in contaminants when compared to untreated diatomite. This treatment renders the diatomite highly conducive for the incorporation of WO_3_, given the increased availability of sites within the porous structure. Fig. 3e and f provides compelling evidence of the successful creation of WO_3_/diatomite composite materials. Here, WO_3_ nanoplates are adeptly integrated into the porous framework of diatomite, filling the voids and interstices effectively. The specific surface areas (*S*_BET_) of raw diatomite, acid-treated diatomite and 10% WO_3_/diatomite were 21.35, 24.92 and 31.34 m^2^ g^−1^, respectively. The *S*_BET_ of the 10% WO_3_/diatomite sample exhibits a more significant increase compared to the acid-treated diatomite, which can be attributed to the presence of smaller-sized WO_3_ particles on the diatomite carrier. The presence of such a porous structure within diatomite is a significant advantage, as it offers a straightforward means of incorporating additional porosity into the material a characteristic of paramount importance in the context of investigations aimed at antibiotic degradation. These observations collectively underscore the potential of this material as a promising candidate for further exploration in the realm of antibiotic degradation studies, benefitting from its intricate porous architecture and the ease with which it can be tailored to specific applications.

**Fig. 4:**
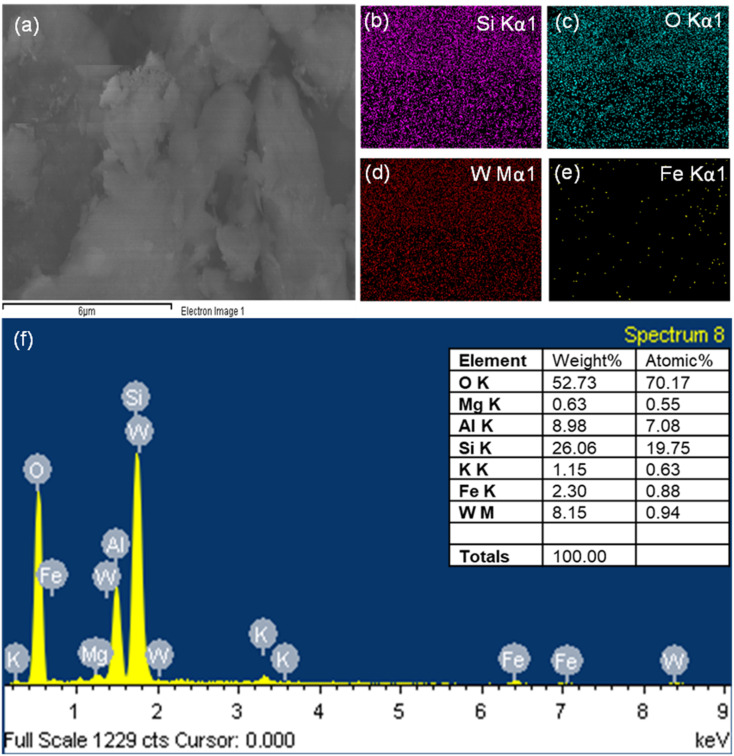
SEM of 10% WO_3_/diatomite (a), elemental mapping (b–e) and EDS spectrum of 10% WO_3_/diatomite (f).

The elemental composition of the 10% WO_3_/diatomite sample is discernible through analysis of the energy-dispersive X-ray spectroscopy (EDX) spectra. As illustrated in Fig. 4, the obvious presence of WO_3_ and its characteristic peaks are conspicuously evident in the 10% composite sample. Once more, the EDX spectrum unequivocally confirms the successful integration of WO_3_ into the diatomite matrix, reaffirming the presence of WO_3_ within the diatomite structure. These findings provide crucial insights into the material's elemental composition, further substantiating its suitability for the intended applications and confirming the effective synthesis of the composite material.

### 3.3. Optical properties

**Fig. 5:**
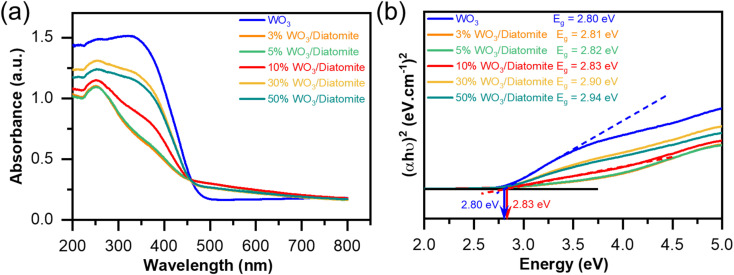
DRS spectra of the as-prepared materials (a) and estimated band gap of WO_3_ and 10% WO_3_/diatomite composite (b).

We have explored the optical properties of WO_3_ and their composite materials using diffuse reflectance spectroscopy (DRS). The DRS spectra of the composite samples with varying WO_3_ concentrations are presented in Fig. 5a. To approximately quantify the band gap energy of these materials, we employ the well-established Tauc plot function (eqn (3)).^39,40^3
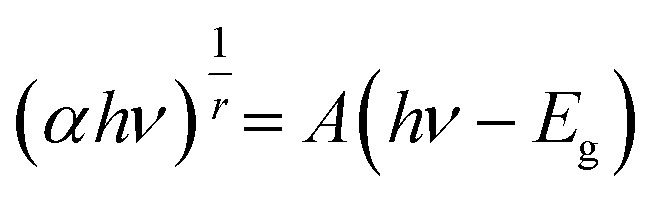
where *α*, *h*, *ν*, *A*, and *E*_g_ represents the absorption coefficient, Planck's constant, the light frequency, a constant, and the band gap energy of the material, respectively; *r* = 2 for indirect transitions.

The obtained band gap (*E*_g_) of WO_3_ is 2.80 eV and the band gap of the composite samples increases from 2.81 to 2.94 eV (Fig. 5b). The increase of the band gap of the composite sample is explained by the existence of SiO_2_ and some the others compounds in the diatomite mineral. The DRS results and band gap values of the 30% WO_3_/diatomite and 50% WO_3_/diatomite composite materials do not exhibit any significant differences, which suggests that the WO_3_ content reaches 30 wt% in the composite, the optical properties of the composites have gradually stabilized. Although there is a slight increase of the band gap energies in the composite samples, the composite samples still maintain the ability to interact with light in the visible region.

**Fig. 6:**
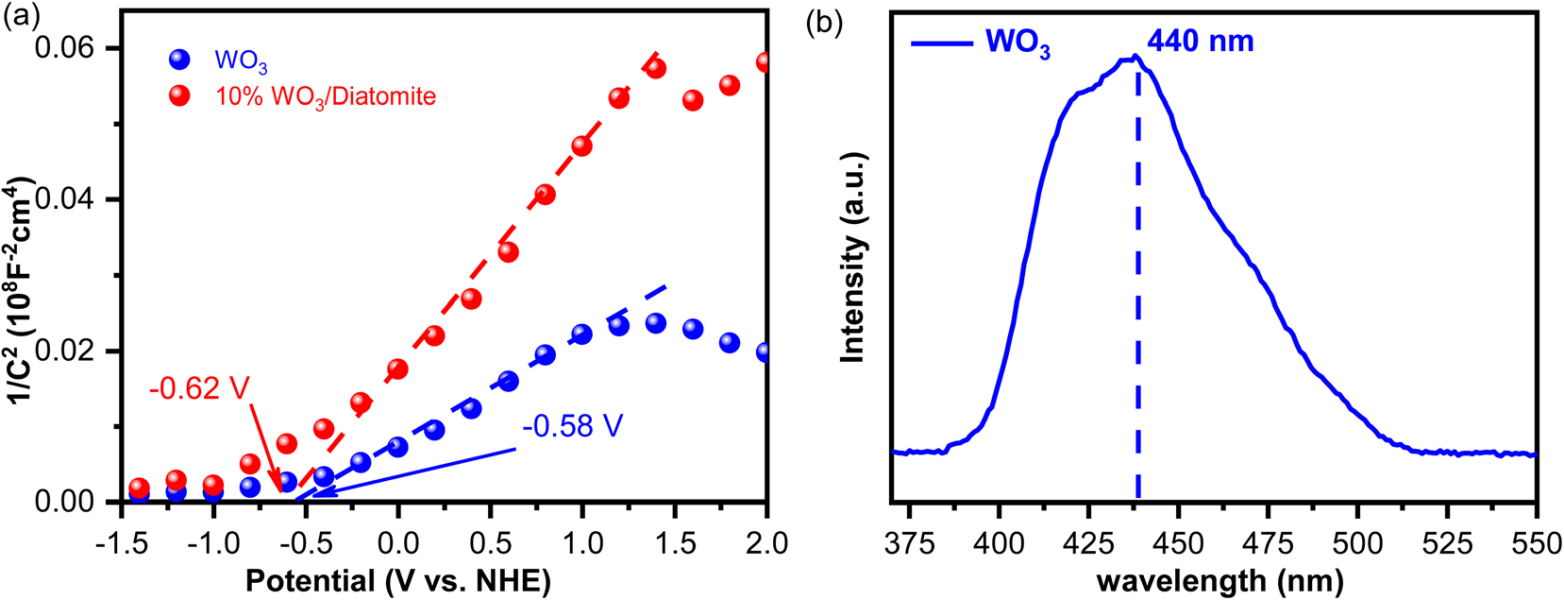
(a) Mott–Schottky plots for WO_3_ and 10% WO_3_/diatomite composite, (b) photoluminescence (PL) spectra of WO_3_ with the excitation wavelength at 300 nm.

To ascertain the electrical characteristics of the materials, we conducted Mott–Schottky plots for both WO_3_ and the 10% WO_3_/diatomite composite samples (Fig. 6). The Mott–Schottky relationship entails the measurement of apparent capacitance as a function of potential under depletion conditions, governed by eqn (4).^41^4
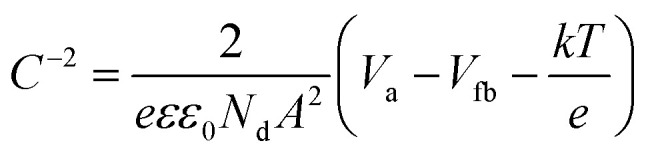
where *C* is the capacitance of the space charge region, *ε* is the dielectric constant of the semiconductor, *ε*_0_ is the permittivity of free space, *N* is the donor density, *A* is the area of the interface or the electrode, *V*_a_ is the applied potential, *V*_fb_ is the flat band potential, *k* is the Boltzmann constant, and *T* is the temperature.

Based on our Mott–Schottky analysis (Fig. 6a), we obtained flat band potentials of −0.62 V *vs.* NHE for WO_3_ and −0.58 V *vs.* NHE for 10% WO_3_/diatomite composite. These values are indicative of the materials' electrical behavior and their potential as semiconductor materials. It is noteworthy that both samples display Mott–Schottky plots with a positive slope, unequivocally establishing their classification as n-type semiconductors. The *V*_fb_ values of both WO_3_ and 10%WO_3_/diatomite are almost equal. This can be attributed to the fact that WO_3_ serves as the primary semiconductor material, while diatomite functions merely as a carrier. Additionally, there is no chemical bonding between WO_3_ and diatomite, which means that the electrical properties of WO_3_ remain unaffected. To confirm the band gap value of WO_3_, a photoluminescence spectrum of WO_3_ nanoparticles was conducted at room temperature using an excitation wavelength of 300 nm (Fig. 6b). The analysis revealed that the WO_3_ exhibited a maximum emission wavelength of 440–442 nm (∼2.80–2.82 eV), which is consistent with the findings from the DRS and Tauc band gap (Fig. 5).

### 3.4. Photocatalytic tetracycline degradation

**Fig. 7:**
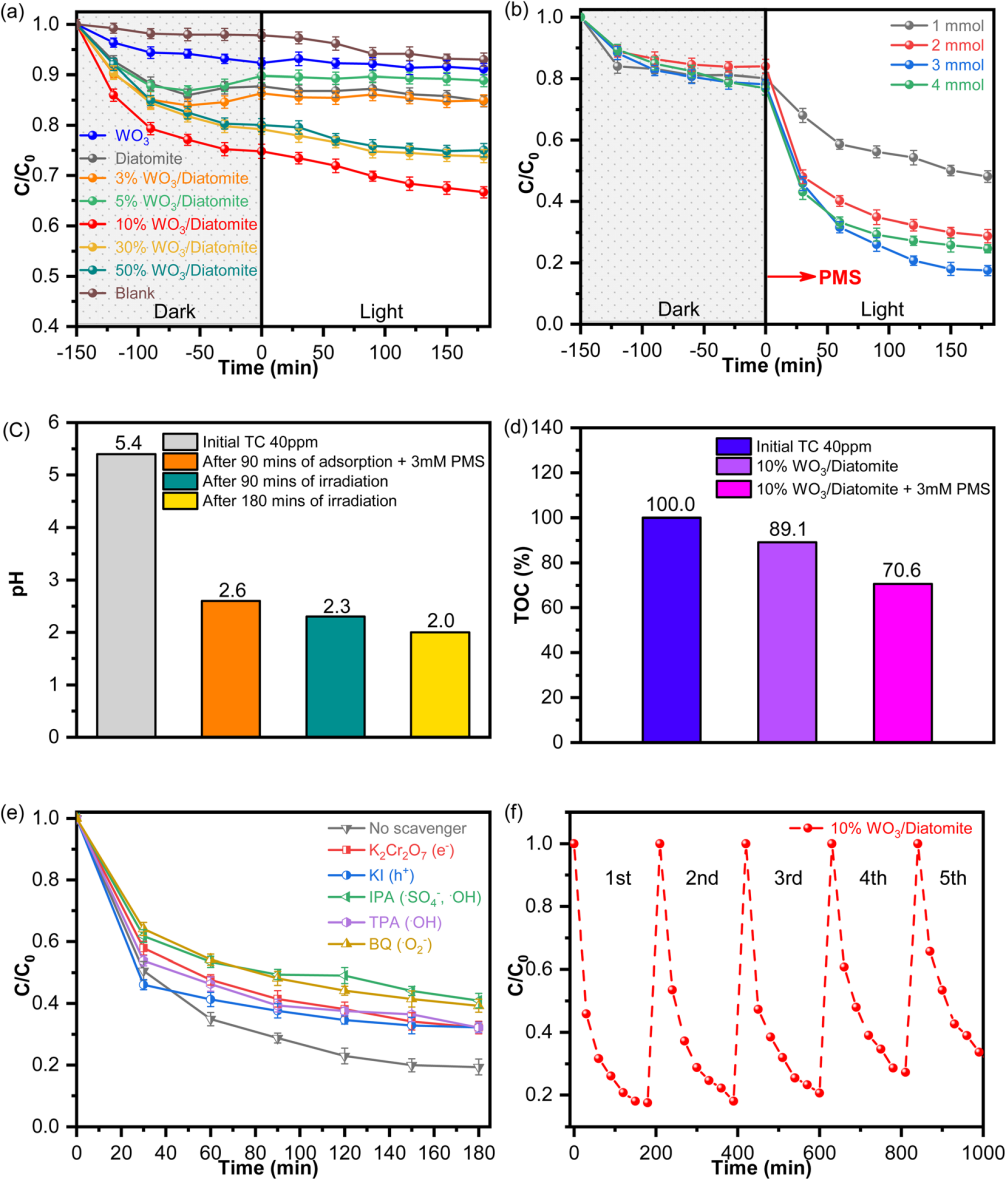
Photocatalytic activity of the materials: (a) photocatalytic tetracycline degradation of the materials under visible light irradiation. (b) Photocatalytic tetracycline degradation of the materials under visible light irradiation with PMS activation. (c) Change of pH value. (d) Tetracycline mineralization. (e) Free radical trapping studies of the 10% WO_3_/diatomite composite, and (f) stability of the 10% WO_3_/diatomite composite for five repeated photocatalytic runs.

To assess the efficacy of photocatalytic antibiotic degradation over the as-prepared materials, we use a tetracycline solution with an initial concentration of 40 mg L^−1^ as the target pollutant for degradation using 200 mg L^−1^ of the catalyst. Prior to activating PMS, a series of preliminary tests are conducted to evaluate the antibiotic degradation capacity of WO_3_, diatomite, and WO_3_/diatomite composites with varying WO_3_ concentration, changing of pH value during in the photocatalytic process (Fig. 7c). This systematic approach enables our understanding about the capability of the materials in removing tetracycline, informing the most effective composition for more in-depth studies.

To reach adsorption/desorption equilibrium, the solution is agitated in the absence of light for 150 minutes. Control samples without a photocatalyst (blank) are analyzed and compared under identical conditions, providing experimental baseline for our assessments. Under light irradiation for 180 minutes, a minor fraction (2%) of the tetracycline solution spontaneously decomposed in the absence of a catalyst. Due to its porous structure, diatomite exhibited superior tetracycline adsorption capabilities compared to the WO_3_ sample. However, the photocatalytic degradation efficiencies for bare WO_3_ and diatomite samples remained relatively low, at 2.34% and 3.41%, respectively. Among the composite samples, 10% WO_3_/diatomite composite achieves the highest degradation efficiency (10.93%) after 180 minutes of irradiation. This underscores the pivotal role of WO_3_ as a key factor for the degradation of antibiotics. Notably, the efficiency diminished as WO_3_ concentration increased to 30% (6.78%) and 50% (6.25%) within the composite material. This decline can be attributed to the excessive presence of WO_3_, which fills the pores in the diatomite, limiting the activation of WO_3_ only on its surface. Furthermore, when diatomite is excessively coated with WO_3_, its function as a carrier diminishes. The degradation kinetics of tetracycline solutions during the tetracycline degradation follow first-order kinetics, as described by eqn (2). As detailed in Table 1, the rate constant for the 10% WO_3_/diatomite photocatalyst after 90 minutes of exposure is calculated to be 0.00398 min^−1^, signifying the sample's superior efficacy in the degradation of antibiotics. These findings underscore the potential of the 10% WO_3_/diatomite composite as an efficient photocatalyst for the degradation of tetracycline. Consequently, the 10% WO_3_/diatomite sample is selected as the optimal catalyst for subsequent experiments.

**Table 1:** Decomposition effectiveness of the samples with varying WO_3_ content after 180 minutes and response rate after 90 minutes

Photocatalyst	Efficiency (%)	Reaction rate (min^−1^)
WO_3_	2.34	0.00091
Diatomite	3.41	0.00151
3% WO_3_/diatomite	2.67	0.00166
5% WO_3_/diatomite	2.33	0.00121
10% WO_3_/diatomite	10.93	0.00398
30% WO_3_/diatomite	6.78	0.00323
50% WO_3_/diatomite	6.25	0.00306

#### 3.4.1. Photocatalytic PMS activation activity

In our investigation of antibiotic degradation using the 10% WO_3_/diatomite composite sample through PMS activation, we use the same conditions as in the previous experiment (a tetracycline solution with a concentration of 40 mg L^−1^ and a catalyst concentration of 200 mg L^−1^). The degradation efficiency as a function of varying PMS concentrations, ranging from 1 mM to 4 mM, is presented in Fig. 7b. Our results suggest that PMS can makes the solution more acidic due to its ability to dissociate H^+^ ion (Fig. 7c) and 3 mM is the optimal PMS concentration for generating the highest amount of ˙SO_4_^−^ radicals conducive for photocatalytic tetracycline degradation. Specifically, a remarkable degradation efficiency of nearly 81% is observed at this PMS concentration. Beyond this PMS concentration (*e.g.*, 4 mM), there is a decline in degradation efficiency, dropping to 67.86%. The observed change in efficiency at elevated concentrations could be due to the overproduction of ˙SO_4_^−^ and ˙OH radicals. These excessive radicals, at higher concentrations, could adversely affect degradation efficiency. Explicitly, they might react with HSO_5_^−^ to form ˙SO_5_^−^ radicals as described in eqn (5) or engage with one another to yield S_2_O_8_^2−^ anions as described in eqn (6). In addition, Table 2 shows that the kinetic constant *k* of the 10% WO_3_/diatomite composite, in which the *k* value for the 3 mM PMS is 0.0416 min^−1^. This result is pertinent to the efficiency degradation of 3 mM PMS in the 10% WO_3_/diatomite composite. The observations elucidate the complexities inherent in the PMS activation process. They emphasize the necessity to finely tune the PMS concentration to attain peak photocatalytic degradation efficiency. It is also evident that steering clear of specific concentrations can avert undesirable side reactions that might impede the degradation process. TOC analysis results (Fig. 7d) also show that in the presence of PMS, the tetracycline mineralization of the 10% WO_3_/diatomite samples is better, meaning that CO_2_ produced in this process is more intense. This result is completely consistent with the mechanism of generating more free radicals with stronger oxidizing properties in this reaction. Moreover, Table 3 shows the strong photocatalytic activity for tetracycline degradation of the 10% WO_3_/diatomite system compared to other recently published materials.

5 ˙SO_4_^−^/˙OH + HSO_5_^−^ → ˙SO_5_^−^ + SO_4_^2−^ + H^+^/OH^−^

6 ˙SO_4_^−^ + ˙SO_4_^−^ → S_2_O_8_^2−^

**Table 2:** The photocatalytic degradation efficiency of tetracycline in molar PMS concentration survey

Concentration of PMS (mM)	Efficiency (%)	Reaction rate (min^−1^)
1	41.3	0.0118
2	65.79	0.0292
3	80.75	0.0416
4	67.86	0.0322

**Table 3:** A comparison of photocatalytic activity related to WO_3_ immobilized on diatomite substrate and photocatalyst degradation tetracycline

Catalyst	Organic contaminant (ppm)	Light source	Catalyst dosage (g L^−1^)	Time (h)	Degradation efficiency (%)	Ref.
TiO_2_/WO_3_/diatomite (T25-W5/Di)	Paraquat/12.6	Solar (400 W)	1.4	3.0	80	24
WO_3_/Fe_3_O_4_/diatomite (WO_3_/Fe_3_O_4_/DT)	Rhodamine B/5	Xenon (500 W)	0.3	0.6	78	42
	Methyl orange/5				95	
	Methylene blue/5				83	
PVDF-TiO_2_@g-C_3_N_4_	Tetracycline/50	Vis (300 W)	1.0	5	97	43
Bi_2_WO_6_/g-C_3_N_4_	Tetracycline/10	Sunlight (35 W)	1.0	1.5	95	44
Ag/Bi_2_Sn_2_O_7_–C_3_N_4_	Tetracycline/20	UV (400 W)	1.0	1.5	89	45
10% WO_3_/diatomite + 3 mM PMS	Tetracycline/40	Vis (300 W)	0.2	3.0	80	This work

#### 3.4.2. Photocatalytic degradation mechanism

To gain a comprehensive understanding of the primary constituents driving the photocatalytic reaction, we conduct a series of free radical trapping studies. These experiments are meticulously designed, wherein we incorporate quenching agents like isopropyl alcohol (IPA), potassium dichcromate (K_2_Cr_2_O_7_), potassium iodide (KI), *p*-benzoquinone (BQ), and *tert* butyl alcohol (TBA) into the 10% WO_3_/diatomite composite sample. Each of these agents was added at a consistent weight percent of 1%, serving the purpose of selectively suppressing different reactive species. The introduction of various quenching agents like K_2_Cr_2_O_7_, KI, TBA, and BQ, which results in degradation efficiencies of 67.85%, 67.78%, 67.85%, and 60.78% respectively, demonstrated only a marginal effect on the photocatalytic degradation efficiency of tetracycline (Fig. 7e). Such observations indicate that while the targeted species by these agents, *i.e.*, e^−^, h^+^, ˙OH, and ˙O_2_^−^, do participate in the photocatalytic activity, they are not the primary contributors to the degradation mechanism. In contrast, the introduction of IPA causes a substantial reduction, bringing the degradation efficiency down to 59.10% (see Table 4). Given IPA's capacity to scavenge both ˙OH and ˙SO_4_^−^ radicals and considering that ˙OH plays a relatively minor role in the photocatalytic degradation, it becomes evident that the ˙SO_4_^−^ radical holds paramount importance in driving the photocatalytic processes.

**Table 4:** The photocatalytic degradation efficiency of tetracycline in trapping test

Quenching agents	Tetracycline removal efficiency (%)
IPA	59.10
K_2_Cr_2_O_7_	67.85
KI	67.78
TBA	67.85
BQ	60.78
No scavenger	80.75

Understanding the sustainable efficacy and longevity of photocatalytic materials is pivotal for their practical applications. With this motivation, we delved into the cyclical degradation performance of the 10% WO_3_/diatomite composite sample in the presence of 3 mM PMS, as illustrated in Fig. 7f. Impressively, the sample's efficiency in degrading tetracycline remained relatively stable for the initial three cycles. Even after five cycles, despite a minor dip in performance, the sample sustained an efficiency of up to 71%. It is noteworthy that during the first 30 minutes of radiation across all five cycles, there was a pronounced decrease, likely attributable to the presence of PMS. This consistent performance, even after multiple cycles, accentuates the sample's remarkable resilience and underscores its potential in tetracycline reduction.

**Fig. 8:**
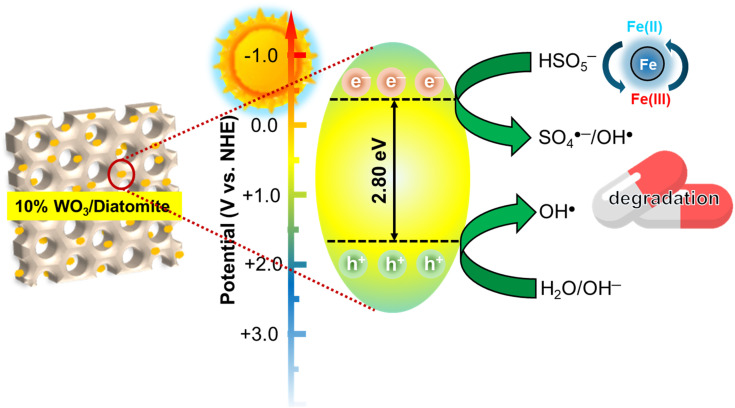
Proposed photocatalytic tetracycline degradation of 10% WO_3_/diatomite composites in coupling with PMS activation under visible light.

Drawing from the comprehensive analysis of our experimental data, we have delineated a potential mechanism, graphically illustrated in Fig. 8. Upon a composite of WO_3_ and diatomite is formed, the newly established WO_3_/diatomite interface gives rise to a reduction in the optical band gap of the composite compared to the bare states. Such an interface also modulates the electron density in the composite and mitigates electron–hole recombination process. Notably, central to this mechanism is the prominent role of the ˙SO_4_^−^ radicals, which emerges as the principal agent driving the degradation of tetracycline in the 10% WO_3_/diatomite composite sample. It is evident from our findings that the activation of PMS can be achieved either *via* e^−^ or ˙O_2_^−^, leading to the formation of ˙SO_4_^−^ radicals (eqn (7)–(13)). This formation enhances the separation efficacy of electron–hole pairs. It is also worth noting that h^+^ possesses the ability to interact with PMS, resulting in the production of ˙SO_5_^−^, a radical with comparatively diminished reactivity than its counterpart, ˙SO_4_^−^ (eqn (14) and (15)). Intriguingly, at elevated concentrations of ˙SO_5_^−^, it has the potential to transform into ˙SO_4_^−^ (eqn (16)). Uniquely, as α-Fe_2_O_3_ is one of the two primary components of diatomite, it is possible to take them into account as iron ions are highly reactive to HSO_5_^−^ to generate SO_4_^2−^ and OH˙ (eqn (17)–(19)). Overall, the results from the free radical trapping test suggest that e^−^ and h^+^ have the capability to produce ˙O_2_^−^ and ˙OH radicals, respectively. However, their roles seem to be supplementary, acting as secondary agents in the comprehensive efficiency of tetracycline decomposition (eqn (20)). This mechanism offers a detailed perspective on the intricate routes inherent in the photocatalytic procedure.

7 10% WO_3_/diatomite + *hυ* → 10% WO_3_/diatomite + h_VB_^+^ + e_CB_^−^

8 O_2_ + e_CB_^−^ → O_2_˙^−^

9 O_2_˙^−^ + H_2_O → OH˙ + OH^−^

10 h_VB_^+^ + H_2_O → H^+^ + OH˙

11 h_VB_^+^ + OH^−^ → OH˙

12 e_CB_^−^ + HSO_5_^−^ →SO_4_˙^−^ + OH^−^

13 e_CB_^−^ + HSO_5_^−^ → SO_4_^2−^ + OH˙

14 h_VB_^+^ + HSO_5_^−^ → SO_5_˙^−^ + H^+^

15 HSO_5_^−^ + O_2_˙^−^ → SO_4_˙^−^ + HO_2_^−^

16 2SO_5_˙^−^ → SO_4_˙^−^ + O_2_

17 HSO_5_^−^ + Fe_(diatomite)_^3+^ → SO_5_˙^−^ + Fe^2+^ + H^+^

18 HSO_5_^−^ + Fe^2+^ → SO_4_˙^−^ + Fe^3+^ + OH^−^

19 HSO_5_^−^ + Fe^2+^ → SO_4_^2−^ + Fe^3+^ + OH˙

20 SO_4_˙^−^/OH˙ + tetracycline → CO_2_ + H_2_O + SO_4_^2−^

## 4. Conclusion

In this study, we have proposed the first development of WO_3_/diatomite composite photocatalysts, marking a significant advancement in the effective removal of antibiotics. This achievement is accentuated when paired with PMS activation under visible light. The enhanced photocatalytic performance in antibiotic degradation can be attributed to the meticulous formation of the composite and the judicious selection of an optimal PMS concentration. Specifically, the 10% WO_3_/diatomite composites, when combined with the ideal PMS concentration, emerge as a robust and enduring photocatalyst. In addition, the WO_3_/diatomite catalyst can degrade 80.75% tetracycline (40 mg L^−1^) after 180 min-visible light irradiations. Furthermore, WO_3_/diatomite/PMS systems are stable (achieved up to 71% of tetracycline degradation performance after five cycles). Delving deep into these mechanisms not only enriches our understanding but also opens avenues for impactful applications in the realm of antibiotic degradation.

## Ethical approval

(1) The article is original. (2) The article has not been published previously. (3) The article is not under consideration for publication elsewhere. (4) No conflict of interest exists, or if such conflict exists, the exact nature must be declared. (5) If accepted, the article will not be published elsewhere in the same form, in any language, without the written consent of the publisher.

## Consent to participate and publish

The article has been written by the stated authors who are ALL aware of its content and approve of participating and publish our submission.

## Data availability

Data available on request from the authors.

## Author contributions

The Luan Nguyen: investigation; funding acquisition. Hong Huy Tran; Tu Cam Huynh; Khoa Tien Le: investigation; Thi Minh Cao: investigation, resource. Viet Van Pham: writing – review & editing, supervision.

## Conflicts of interest

There is no interest to declare.
